# Relationship between lysine methyltransferase levels and heterochromatin gene repression in living cells and in silico

**DOI:** 10.1093/pnasnexus/pgad062

**Published:** 2023-03-07

**Authors:** Xiaokang Yan, Michael R Williams, Ameriks D Barboza Castillo, Dmitri Kireev, Nathaniel A Hathaway

**Affiliations:** Center for Integrative Chemical Biology and Drug Discovery, Division of Chemical Biology and Medicinal Chemistry, UNC Eshelman School of Pharmacy, University of North Carolina, Chapel Hill, NC 27599, USA; Center for Integrative Chemical Biology and Drug Discovery, Division of Chemical Biology and Medicinal Chemistry, UNC Eshelman School of Pharmacy, University of North Carolina, Chapel Hill, NC 27599, USA; Center for Integrative Chemical Biology and Drug Discovery, Division of Chemical Biology and Medicinal Chemistry, UNC Eshelman School of Pharmacy, University of North Carolina, Chapel Hill, NC 27599, USA; Department of Chemistry, University of Missouri—Columbia, Columbia, MO 65211, USA; Center for Integrative Chemical Biology and Drug Discovery, Division of Chemical Biology and Medicinal Chemistry, UNC Eshelman School of Pharmacy, University of North Carolina, Chapel Hill, NC 27599, USA

**Keywords:** heterochromatin formation, mouse stem cells, SETDB1, Monte Carlo simulation model, dCas9-KRAB

## Abstract

Gene regulation plays essential roles in all multicellular organisms, allowing for different specialized tissue types to be generated from a complex genome. Heterochromatin-driven gene repression, associated with a physical compaction of the genome, is a pathway involving core components that are conserved from yeast to human. Posttranslational modification of chromatin is a critical component of gene regulation. Specifically, tri-methylation of the nucleosome component histone 3 at lysine 9 (H3K9me3) is a key feature of this pathway along with the hallmark heterochromatin protein 1 (HP1). Histone methyltransferases are recruited by HP1 to deposit H3K9me3 marks which nucleate and recruit more HP1 in a process that spreads from the targeting site to signal for gene repression. One of the enzymes recruited is SETDB1, a methyltransferase which putatively catalyzes posttranslational methylation marks on H3K9. To better understand the contribution of SETDB1 in heterochromatin formation, we downregulated SETDB1 through knockdown by a dCas9-KRAB system and examined heterochromatin formation in a chromatin in vivo assay (CiA-Oct4). We studied the contribution of SETDB1 to heterochromatin formation kinetics in a developmentally crucial locus, *Oct4*. Our data demonstrate that SETDB1 reduction led to a delay in both gene silencing and in H3K9me3 accumulation. Importantly, SETDB1 knockdown to a ∼50% level did not stop heterochromatin formation completely. Particle-based Monte Carlo simulations in 3D space with explicit representation of key molecular processes enabled the elucidation of how SETDB1 downregulation affects the individual molecular processes underlying heterochromatin formation.

**Significance Statement**

Heterochromatin formation is an essential biological process involved in regulating gene expression and genome integrity. SETDB1 is a key enzyme acting to establish and maintain heterochromatin gene repression. Here, we examine the kinetics of heterochromatin in detail at a single genetic locus, to discern the role of SETDB1 in histone methylation and gene repression. We created SETDB1 knockdown cell lines through dCas9-KRAB and compared heterochromatin formation kinetics under such condition. Moreover, to further discern the roles of individual molecular players and investigate experimentally unobservable processes, such as the spread of distinct H3K9 methylation marks, we investigated the methylation process in a 3D computer simulation at submolecular resolution.

## Introduction

The mammalian genome is copied with high fidelity through cell division passing on abundant information to the next generation of cells. Through proper regulation of this information, cells within the same organism can have a variety of shapes with distinct functions. This regulation is achieved through multiple pathways including methylation of CpG islands, covalent chemical modifications placed on histone proteins, and other epigenetic regulatory mechanisms (1–3). These chemical modifications signal for changes in chromatin accessibility and gene expression profile (3–5). Euchromatin is associated with the accessible, transcriptionally active regions of the genome, while heterochromatin is associated with less accessible, silenced regions of the genome (6). Each chromatin state associates with various epigenetic modifications, including posttranslational modifications (PTMs) on histone proteins. Importantly, for the focus of this work, histone protein modification state is passed on from generation to generation as a form of epigenetic information. PTMs on histone proteins can be on multiple amino acid positions, but there are many PTMs added in the *N*-terminal regions of histones (sometimes called histone tails). Among the histone PTMs, histone methylation is a prolific signaling mark present in either euchromatin or heterochromatin, depending on its PTM context. Histone PTMs along with corresponding chromatin states are faithfully passed down during cell replication to maintain the cell expression profile defining any given tissue. Aberrant epigenetic marks that favored oncogenic effects are often observed in cancer cells and can play a driving role in disease pathology (7–10).

Heterochromatin formation is involved in a number of important cellular functions. During mammalian development, heterochromatin formation helps to restrict the cell's potential and silence pluripotency genes such as *Nanog*, *Oct4*, and *Sox2* (11–16). Conversely, the removal of heterochromatin marks also initiates the expression of tissue-specific genes (13). Heterochromatin formation is also necessary for the formation of centromere and maintenance of telomere length (17). By silencing endogenous retrovirus expression, heterochromatin also protects genome from unwarranted recombination (18–20). Thus, the role of heterochromatin in development and genome maintenance of the mammalian organisms is vast.

The accumulation of histone H3 lysine 9 tri-methylation (H3K9me3) (21, 22) is an important mark of the heterochromatin state (23–25). H3K9me3-mediated heterochromatin formation involves heterochromatin protein 1 (HP1) that contains a chromodomain that recognizes and binds to di- and tri-methylated H3K9 (25–29). HP1 also contains a chromoshadow domain (CSD) which has dual functions during heterochromatin formation. First, the chromoshadow domain has propensity to dimerize with other chromoshadow domains thus promoting chromatin condensation through nucleosome bridging (30–33). Second, it can recruit chromatin-modifying enzymes, SUV39H1/2 (34, 35), SETDB1 (35, 36), and G9a (35, 37), belonging to the SET family of histone methyltransferases (HMTs) (38), to further spread H3K9me3 marks. Previous studies showed that the three HMTs are not equivalent or interchangeable. SETDB1 and SUV39H1/2 are both responsible for the di- and tri-methylation (39, 40), while G9a catalyzes the initial mono-methylation of H3K9 (37, 41). However, the exact functions of each specific HMT are yet to be understood as well as other mechanisms in heterochromatin formation. It is still unclear whether any of them is dispensable for heterochromatin formation. Some reports show that the downregulation of SETDB1 and SUV39H1/2 is associated with the decrease of H3K9me3 level and HP1 recruitment to chromatin (19, 39, 42–45). It was also reported that, unlike SUV39H1/2, SETDB1 localizes to both heterochromatin and euchromatin regions, suggesting that transitions from active euchromatin to repressed heterochromatin requires SETDB1 (46, 47).

Previous work has demonstrated that SETDB1 disruption can lead to the gene upregulation and changes in the chromatin state; however, we do not know how SETDB1 disruption led to a failure of heterochromatin formation or gene regulation. In our previous work, we have combined biological and computational simulation experiments to understand the HP1α-induced heterochromatin formation process (33). This work extends our previous work to probe the contribution of SETDB1 in HP1α-induced heterochromatin formation using both experimental and computational methods.

In order to probe SETDB1's contribution to heterochromatin formation, we knocked down SETDB1 expression levels and examined the impact of the loss of this histone methyltransferase. Since the discovery of the CRISPR-Cas9 system, genetic alteration has become much easier and more straightforward in multiple cell types. In addition to sequence alterations, CRISPR interference (CRISPRi) has been used as an alternative to siRNA technology to change targeted gene expression (48, 49). Fusing a transcriptional repressor KRAB domain to dCas9, a nuclease-deactivated (dead) Cas9 protein, improved gene repression ability compared with CRISPRi approaches (48–51). In this work, we knocked down SETDB1 using a dCas9-KRAB repression system and examined the changes in heterochromatin formation as measured by gene expression as well as deposition of the hallmark H3K9me3 modification on chromatin.

We utilized the chromatin in vivo assay (CiA-Oct4), to study the process of heterochromatin formation under the SETDB1 knockdown conditions. The CiA-Oct4 system substituted a nuclear GFP in single *Oct4* allele in mouse stem cells. Under stem-cell conditions, enhanced green fluorescent protein (eGFP) would be expressed. Upstream of the eGFP within the *Oct4 locus*, there are two DNA-binding arrays that can be bound by an FK506-binding protein 12 domain (FKBP) fused to either a zinc finger–binding protein or Gal4 DNA-binding protein. These domains are reserved for recruitment of epigenetics modifiers through chemical-induced proximity (CIP). A chimeric protein of HP1α chromoshadow domain (csHP1α) fused with FKBP-rapamycin-binding domain (FRB) is also introduced into the cell. A CIP molecule, rapamycin, can connect the FRB to the FKBP and bring csHP1α to the promoter in front of the eGFP gene reporter at the eGFP reporter at the *Oct4* site. Once tethered to the promoter, csHP1α would initiate the process of heterochromatin formation and therefore silence the eGFP expression. Previous studies on these cell lines have detailed the generation of a heterochromatin domain in response to HP1 recruitment, demonstrating silencing of eGFP coupled with accumulation of heterochromatin marks along with reduction of euchromatin marks. Others have used this cell line to identify heterochromatin formation inhibitors in a high-throughput screen (52) or compounds that facilitate induced pluripotent stem-cell generation (53) and to determine the role of DNA methylation on epigenetic memory (54).

To further discern the molecular processes underlying heterochromatin formation, we investigated the H3K9 methylation process in a 3D computer simulation at submolecular resolution. Previously, computational simulations proved successful in modeling various aspects of the chromatin structural transformations (55–64), although most of the reported models were tuned to simulate mega-base systems (55, 58–60, 65). Recently, we introduced a Monte Carlo particle-based model to a submolecular detail in order to more accurately reflect the entropic burden affecting the dynamics of heterochromatin formation (33). Despite neglecting important aspects of the system's energetics, the model was able to provide a comprehensive spatial and temporal characterization of the heterochromatin formation process suggesting that heterochromatin formation is a largely entropy-driven process. The temporal aspect was enabled through the model parameterization using time- or rate-related evidence. Diffusion data for a diverse set of proteins in cell lysates (66–68) were exploited to calibrate protein motions and validated on an independent set of diffusion measurements for chromatin-binding proteins (69, 70). Kinetics of the protein association and dissociation was assessed from residence times and rates for chromatin-binding proteins (30, 70, 71). Here, we extend the scope of the previous model by enabling enzyme-mediated chemical transformations, i.e. depositing and removing methyl marks at H3K9. The model parameters were derived from experimental data on enzymatic activities of G9a, SUV39H1/2, and SETDB1 and on their binding to HP1 and chromatin. This study offers an insight into the time course of mono-, di-, and tri-H3K9 methylation by each individual HMT that is unprecedented for either computational or experimental techniques. The induced downregulation of G9a, SUV39H1/2, and SETDB1 in the in silico system produced the effect consistent with that observed in chromatin in vivo assay experiments.

## Results

### Generation of SETDB1 knockdown cell lines

**Fig. 1.**
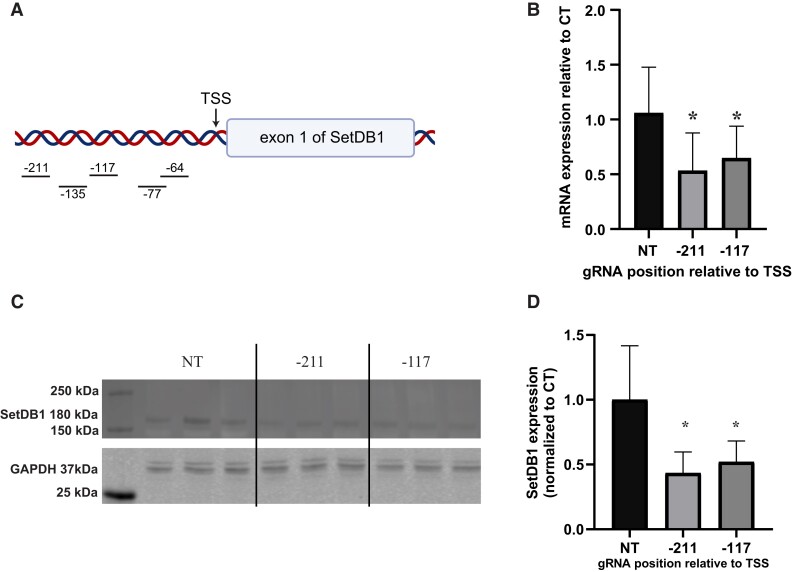
Knockdown of SetDB1 with dCas9-KRAB. A) Schematic representation of the genomic location of SETDB1 targeting gRNAs. B) Quantification of SETDB1 mRNA levels using quantitative PCR for the two most effective gRNA used along with a control nontargeting gRNA. The statistical analysis was done using Student’s t test with two biological repeats and three technical repeats, *N* = 6, * *P* ≤ 0.05. C) Western blot against SETDB1 and GAPDH proteins, conducted in biological triplicate for all samples. D) Graphical representation of SETDB1 Western blot. The statistical analysis was done using Student’s t test with three biological repeats and two technical repeats, *N* = 6, * *P* ≤ 0.05.

In order to examine the contribution of SETDB1 to heterochromatin formation, we modulated its levels using the dCas9-KRAB repression system. SETDB1 expression can be knocked down through stable infection of dCas9-KRAB and gRNAs targeting the *SETDB1* gene. We first infected dCas9-KRAB into the CiA-Oct4 cell line and antibiotic resistance selected for multiple days to ensure that all surviving cells would contain the dCas9-KRAB system. We identified five gRNA sequences near SETDB1 transcription start sites to maximize chance of gene repression (Fig. 1A). Each of gRNA plasmids was stably infected into the cells already infected with dCas9-KRAB and a control gRNA with nontargeting sequence (NT). First, we screened for SETDB1 mRNA expression level within the cells and found two out of the five gRNAs showed significant reduction (Figs. S1 and 1B). A Western blot experiment also confirmed the knockdown of the SETDB1 protein (Fig. 1C and D).

### SETDB1 knockdown impaired HP1α-induced gene silencing

**Fig. 2.**
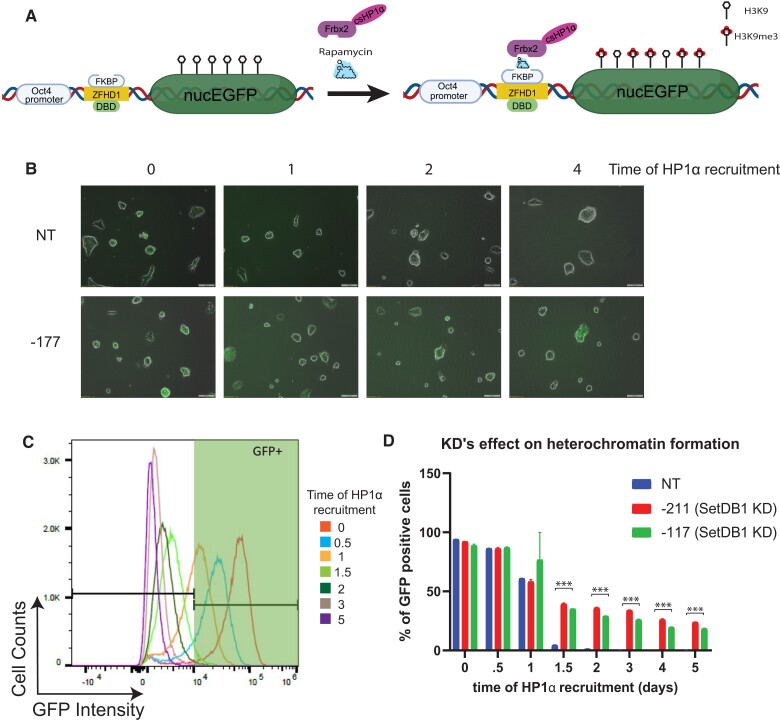
Chemical-induced proximity (CIP) recruitment of HP1α to CiA-Oct4 eGFP allele. A) Schematic representation of CiA-Oct4 eGFP allele. When the CIP-rapamycin is introduced, FRB-HP1α fusion protein is recruited to the promoter region of the eGFP loci; this initiates the heterochromatin process and silences eGFP expression. B) Fluorescent microscope images of NT and one of the knockdown cells lines with images taken at different HP1α recruitment times. Phase channel and eGFP channel are overlaid; see Supplemental Fig. S3 for individual channels. C) Flow cytometry analysis of NT cell lines with CIP-HP1α recruitment times as indicated. D) Quantitated representations of NT and two knockdown cell lines with eGFP+ cell population over HP1α recruitment time. Both of the knockdown cell lines showed significant differences compared with the NT cell lines post 1 day of HP1α recruitment time using Student’s t test with three biological replicates, *N* = 3, ***P ≤ 0.001.

After the establishment of SETDB1 knockdown cell lines, we sought to examine the changes in heterochromatin formation using CiA-Oct4 cell lines. Within the CiA-Oct4 cell line, one allele of *Oct4 loci* is replaced with a nuclear eGFP as a reporter, reflective of gene expression level in this line. Upstream of eGFP, two DNA-binding domains were inserted to one allele of the endogenous *Oct4* promoter. Under normal stem-cell conditions, the *Oct4* promoter will drive the expression of eGFP. One of the DNA-binding domains is a zinc finger–binding array (ZFHD1) that can bind the matching ZFHD1 protein fused with a 12-kDa FKBP serving as an anchor protein for CIP systems. To recruit the repressor, two repeats of FRB of mTor were fused with csHP1α. When we introduce rapamycin, it bridges FKBP and FRB thus tethering csHP1α to the *CiA-Oct4* loci through CIP. Once bound, csHP1α silences eGFP expression through recruitment of endogenous heterochromatin machinery including HMTs. Throughout a 5-day time course experiment, we tracked the heterochromatin formation process as measured by the disappearance of eGFP signal along with the gain of H3K9me3 and examined the effect of SETDB1 knockdown on this process. During the HP1α recruitment, all cells showed similar morphology and maintained stem cell–like characteristics (Figs. 2B and S3).

We characterized the eGFP levels under embryonic stem (ES) condition as an eGFP+ population and then tracked the decrease of the eGFP+ cell population over time (Figs. 2C and S2C). During the early recruitment period, the knockdown cell lines showed a similar repression profile (Fig. 2D). However, there is a dramatic measurable shift in eGFP signal starting at 1.5 day of HP1α recruitment, more pronounced in NT cell lines, while the knockdown cell lines showed a more gradual change (Fig. 2D). While the NT cell line showed a major shift in gene expression by day 2, the knockdown cell line showed a slower change over time (Figs. 2D and S2). The SETDB1 knockdown impaired the HP1α-induced heterochromatin formation and changes the gene repression kinetics.

### SETDB1 knockdown led to a reduction in H3K9me3 accumulation

**Fig. 3.**
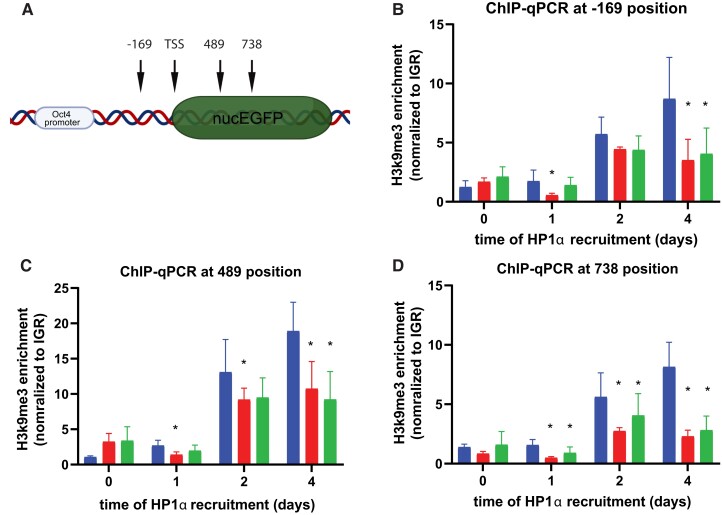
Chromatin immunoprecipitation of H3K9me3 following CIP-HP1α recruitment to CiA-Oct4 eGFP allele. A) PCR primer locations within the *CiA-Oct4* eGFP loci. B–D) H3K9me3 enrichment at −169, 489, and 738 positions relative to the transcriptional start site (TSS) at different CIP-HP1α recruitment times as indicated. Student’s t test with two biological replicates and four technical repeats, *N* = 8, * *P* ≤ 0.05.

In addition to eGFP repression, we examined how SETDB1 knockdown affects the H3K9me3 spread in the proximity of the HP1 recruitment site, as this is a key molecular signal of heterochromatin. During the endogenous heterochromatin formation process, H3K9me3 accumulation is both a product and a player. HP1 protein binds to H3K9me3 through its chromodomain and then recruits epigenetic modifiers to deposit more H3K9me3 to, in turn, recruit more HP1 to further promote the spread of the heterochromatin state. Here, we performed chromatin immunoprecipitation followed by quantitative PCR (ChIP-qPCR) to directly measure the accumulation of H3K9me3 over time. We performed ChIP-qPCR against H3K9me3 after 0, 1, 2, and 4 days of HP1α recruitment. Three qPCR primers are scattered throughout the eGFP gene (Fig. 3A) to measure the H3K9me3 accumulation during the heterochromatin formation. We found at positions +489 from the TSS within the eGFP gene body had the highest level of H3K9me3 accumulation.

All three cell lines showed an increase in H3K9me3 accumulation over HP1α recruitment especially 2 days following HP1α recruitment which is similar to the time we see substantial changes in eGFP expression. The SETDB1 knockdown cell lines showed similar jump to H3K9me3 accumulation starting day 2, but at a reduced level compared with the wild-type (WT) lines with the NT control. With reduced H3K9me3 accumulation, the heterochromatin silencing is less effective complementing what we saw with the eGFP expression. The reduction of SETDB1 led to a reduction in H3K9me3 accumulation within the Cia-Oct4 loci during the formation of heterochromatin, and we observed a reduction in gene silencing efficiency.

### Probing the effect of SETDB1 knockdown in a particle-based Monte Carlo simulation model

**Fig. 4.**
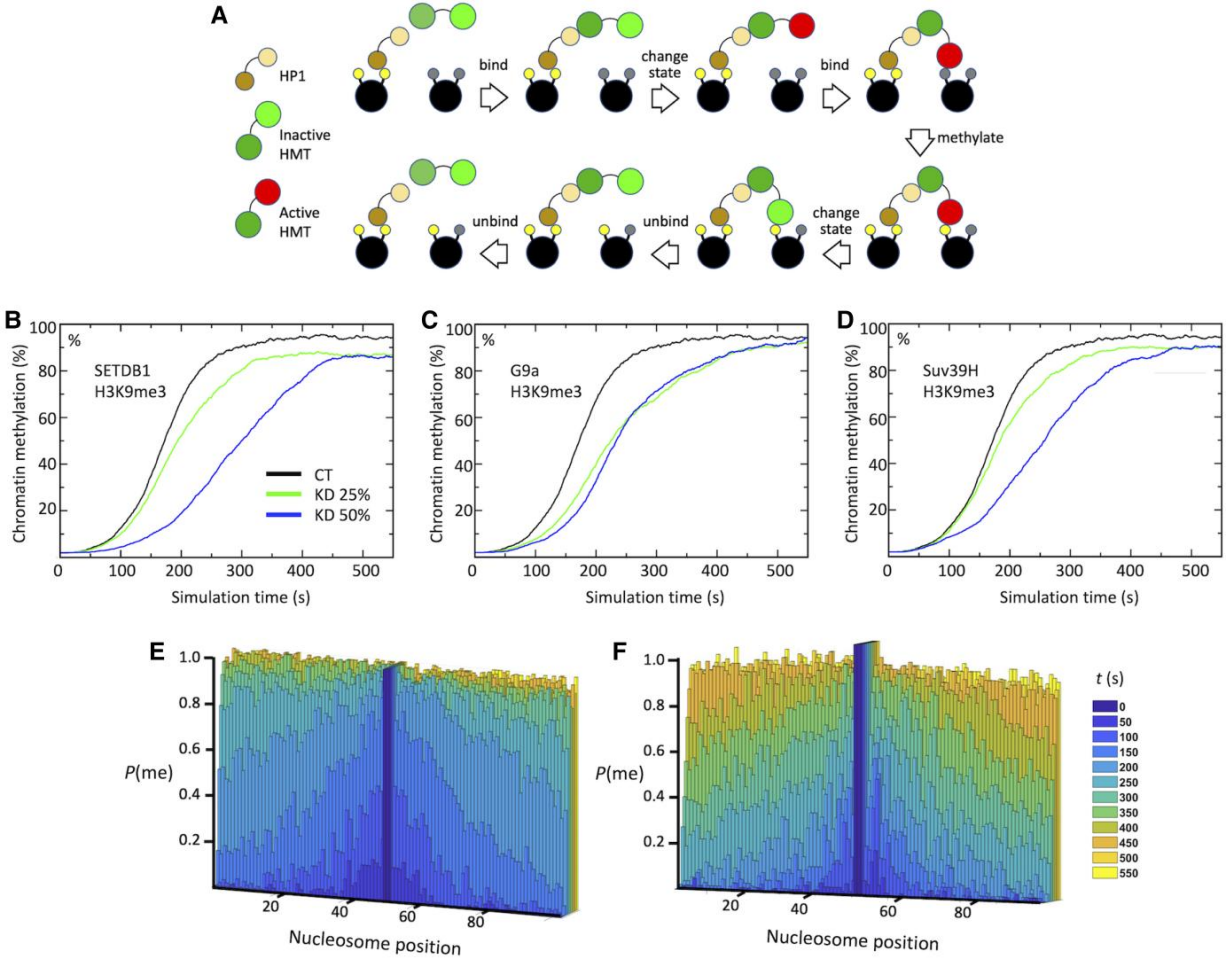
Monte Carlo simulation of individual HMTs’ contribution. A) Molecular objects, their interactions and transformations constituting the histone methylation process; B–D) Time charts of the averaged (over nucleosomes and ensembles) H3K9me3 levels in CT and SETDB1 (B), G9a (C), and Suv39H (*D*) knockdown systems; E, F) Color-coded, ensemble-/time-averaged probability density distributions of H3K9me3 marks over the ∼20-kb chromatin fiber (102 nucleosomes) for the CT (*E*) and SETDB1 50% knockdown systems (*F*).

To gain a mechanistic insight at molecular resolution into H3K9me3 propagation in control lines (CT) and SETDB1 knockdown cells, we made use of our previously described Monte Carlo simulation model (33). The simulated system included a chromatin fiber approximately matching the size of the *Oct4* promoter region (20 kb), HP1, the three known H3K9 methyltransferases (G9a, SETDB1, and SUV39H), and a generic demethylase. The methylation event occurs in a stepwise fashion with the methyl mark deposition by a methyltransferase conditional upon its binding to a chromatin-bound HP1 (Fig. 4A). Methylation (or demethylation) event occurs with a probability derived from experimentally determined enzyme catalytic rates (72–77) when an H3K9 particle (i.e. the substrate) encounters a HMT or a demethylase (see Materials and Methods for details).

In our simulations, the SETDB1 knockdown caused the most significant effect on heterochromatin formation both in terms of the rate of H3K9me3 spread and in a permanent decrease of H3K9me3 level throughout the 10-kb promoter region (Fig. 4B) compared with that for G9a and SUV39H1/2 (Fig. 4C and D). Interestingly, the methylation slowdown resulting from the 25% SETDB1 knockdown system was quite insignificant but produced a ∼10% decrease in the maximum methylation level. At the same time, the 50% SETDB1 knockdown caused a dramatic methylation slowdown (more significant than in any other component knocked down in this system), but no additional decline in the equilibrium methylation level (compared with the 25% knockdown). In fact, SETDB1 was the only HMT in our simulations whose knockdown resulted in a sizable change in the equilibrium methylation level. Apparently, the deficient middle link in the methylation chain (i.e. H3K9me2, mainly produced by SETDB1) exposes the methylation landscape to demethylases to a greater extent than the lack of two other marks and is harder to make up for through secondary enzymatic activities of G9a and SUV39H.

We also explored how SETDB1 knockdown affects H3K9me3 spread at a nucleosome resolution. To this end, we visualized the time-/ensemble-averaged probability density functions of an H3K9me3 mark at each individual nucleosome at several stages of the spread process in WT and 50% knockdown systems (Fig. 4E and F, respectively). One notable observation from these data is that, in agreement with experimental data (25), methylation marks spread from the center toward the ends of the fiber, so that at any given moment, more central nucleosomes have a higher likelihood to be methylated. To our best knowledge, this is the first unbiased particle-based model of chromatin methylation where such a centrifugal effect is observed (even though, in this model, a methylation mark may be deposited on any nucleosome at any stage of the methylation process). Previous computational models of H3K9 methylation made use of a built-in centrifugal spread (78, 79). Submolecular resolution of our model allows to account for the entropic factors linked to the HP1-mediated mechanism of the HMTs binding to chromatin thus preventing the uniform methylation spread. Moreover, it appears that the entropic penalty on methylation of peripheral nucleosomes might be even higher in a SETDB1 knockdown system. As can be seen in Fig. 4F, in that system, methylation is particularly lagging far from the center. For example, in the middle of the process (green bars), the whole fiber reached its maximum methylation level, while only very few near-central nucleosomes achieved the maximum level (the peripheral nucleosomes still have ∼0.5 probability to be methylated).

## Discussion

In this work, we combined biological experimental data with computer simulations to better understand the contribution of HMTs to HP1-induced heterochromatin formation. Previous studies demonstrated that H3K9 methyltransferases and HP1α associate to form complexes, and biochemical assays demonstrated how SUV39H and HP1 chromodomain (CD) work together to spread H3K9me (36, 39, 80). Functional studies of H3K9 methyltransferases mostly reported on global changes to H3K9me3 accumulation and gene upregulation (14, 19, 81), i.e. effects resulting from collective effort of HMTs and demethylases. Yet, little is known about the individual HMTs’ contributions to the dynamic heterochromatin formation process. Here, we examine the kinetics of heterochromatin in detail at a single genetic locus, to discern the role of SETDB1 in histone methylation and gene repression. We created SETDB1 knockdown cell lines with a dCas9-KRAB method and compared heterochromatin formation kinetics under such condition. In addition to the H3K9me3 accumulation and gene transcription, our data demonstrated that SETDB1 knockdown slowed down the kinetics of heterochromatin formation thus resulting in a failure to silence. In the initial HP1α recruitment stage, the difference in eGFP silencing and H3K9me3 accumulation between the control and the knockdown cell lines is negligent. After 1.5 day of HP1α recruitment, eGFP expression was quickly silenced in the control cell lines, while the SETDB1 knockdown cells showed only a gradual reduction in eGFP levels. Along with the overall reduction of H3K9me3 accumulation within the *CiA-Oct4* loci, the reduction of SETDB1 weakened the condition for a strong positive feedback loop between H3K9me3 and HP1 and led to a slower heterochromatin formation.

To further discern the roles of individual molecular players and their microscopic dynamics, we observed the H3K9 methylation process in a 3D computer simulation at submolecular resolution. In these simulations, we can investigate experimentally unobservable processes, such as the effect of each specific HMT on distinct methylation marks (mono-, di-, and tri-methyl) at individual nucleosome positions. For instance, the simulated control system (100% level of all HMTs’ concentrations) showed a rapid increase in the fiber tri-methylation similar to the experimentally observed all-or-nothing behavior. Due to the intricate interplay of three interdependent and partially interchangeable HMTs, the decrease in the tri-methylation rate with the decrease in SETDB1 concentration is not linear. For instance, in the 50% SETDB1 knockout system, the H3K9me3 accumulation kinetics was slowed down dramatically due to a significant shortage of dimethyl marks, whereas the 25% knockdown system closely resembled the control system. A reduction in the ability of the knockdown systems to methylate the entire fiber emerges then from a reduced collective capacity of HMTs to compete with the demethylation by KDMs. These data suggest that the reduction in both eGFP silencing and H3K9me3 level was associated with a buildup of the H3K9me1 mark. Collectively, and somewhat counterintuitively, our data (including the in silico knockdowns of G9a and SUV39H1/H2; Supplementary Fig. S4) suggest that SETDB1 knockdown has the most profound effect on the H3K9 methylation spread. On the whole, our biological experimental observations along with the simulation data demonstrated the essential role of SETDB1 in HP1α-induced heterochromatin formation. In the future, this combined strategy can be broadened to exploring other structural and molecular transformations of the chromatin fiber.

## Materials and methods

### Cell culturing and cell lines

Mouse ES cells were grown on gelatin-coated plates without feeder cells in DMEM supplemented with 4.5 g L^−1^ glucose (Corning), 15% fetal bovine serum, l-glutamate, sodium pyruvate, HEPES buffer (Gibco), NEAA (Gibco), 2-mercaptoethanol (Sigma Aldrich), leukemia inhibitory factor, and penicillin/streptomycin (Gibco) at 37°C supplemented with 5% CO_2_. For the time course experiment, NT and KD cell lines were plated in 80,000 cells per well in a 6-well plate. A 10-μM rapamycin stock dissolved in ethanol was added to media at 3  nM concentration for the time course experiment.

### dCas9 plasmids

dCas9-KRAB plasmid was obtained from Addgene (99372) (50). gRNA plasmid is also from Addgene (64710). Twenty-nmer target sequences were listed in Supplementary Table S1. gRNA plasmids were linearized with BsmBI and ligated with T4 DNA ligation. gRNA sequences were checked with U6 primer.

### Lentiviral infection

A total of 293 T LentiX cells (Clontech) were used to generate lentivirus. The 293TX cells were transfected using polyethyleneimine with lentiviral constructs and gene of interest according to a previously described protocol (82). Post 48 h infections, ES cells were selected according to the plasmid resistance with either blasticidin (InvivoGen), puromycin (InvivoGen), or zeoymycin (InvivoGen). After 1 week of selection, infected cells were used for experiments.

### mRNA extraction and analysis

One million of ES cell lysates were collected for each sample. Cell mRNAs were collected using RNeasy mini kit (Qiagen). Reverse transcription qPCR was performed using Power SYBR Green RNA to Ct 1 step kit (Applied Biosystems). Three biological repeats of each data point were analyzed using ΔΔCt method and normalized against GAPDH. Primer sequences are listed in Supplementary Table S2. The statistical significances were generated using Student’s t test on Excel, and the graphs were generated with Prism (GraphPad Software, Inc.).

### Western blotting

Protein lysates were collected using M-PER mammalian protein extraction reagent (ThermoFisher) with the addition of protease inhibitor (Active Motif) and benzonase (Sigma Aldrich). 15–20 μg of cell lysates were prepared for each Western blot. Protein samples were prepared with 2× Laemmli loading buffer (Bio-Rad) with addition of 2-mercaptoethanol (Sigma Aldrich) and boiled for 5 min at 95°C. Samples were loaded on a 4–20% tris-glycine gradient gels (Bio-Rad) and ran at 200 V for 30 min. Gels were transferred to Immobilon-FL polyvinylidene fluoride (PVDF) membranes (Millipore). PVDF membranes were blocked with blocking buffer (LiCor Odyssey) for an hour. SETDB1 primary antibody (Invitrogen, MA5-15721) and GAPDH primary antibody (AbChem 9485) were added at 1:1,000 dilution and incubated overnight on with rocking motion in 4°C. PBST wash was conducted four times the next day and then incubated with IRDye 680 anti-mouse and IRDye 800 anti-rabbit (LiCor) at 1:15,000 dilution for an hour. Another four-time PBST wash was conducted prior to imaging using Odyssey (LiCor) scanner and analyzed using Image Studio V5.2.

For Western analysis, three biological repeats of the cell lysates were collected, and two technical repeats of the Western were conducted. The SETDB1 intensity was normalized against GAPDH and compared with the NT cell lines. The statistical significances were generated using Student’s t test on Excel, and the graphs were generated with Prism (GraphPad Software, Inc.).

### Flow cytometry

Flow cytometry analysis of eGFP expression was conducted using Attune Nxt machines (ThermoFisher). Three biological replicates of each sample time point of the NT and SETDB1 KD cell lines were collected, and standard deviation was used to report error. Individual samples were cultured in one well of a six-well plate. Individual samples were resuspended in 300 µL of FACs buffer (1×PBS, 1% FBS) then and run on the Attune NXT machine. Flow cytometry data were analyzed using FlowJo software. Cells were gated based on SSC vs. FSC, then FSC-H vs. FSC-A. After gating for single cells, population was separated based eGFP expression. EGFP+ population is based on NT cell line eGFP signal without the recruitment of HP1α as seen in Fig. S2. Graphs were generated with Prism (GraphPad Software, Inc.).

### ChIP sample preparation and RT-qPCR****

Chromatin immunoprecipitation was performed using a previously described protocol (54). Five to ten million ES cell lysates were collected and cross-linked with 1% of formaldehyde for 10 min. Each sample was sonicated in Covaris shearing buffer (0.1% SDS, 1 mM EDTA pH 8, 10 mM Tris HCl pH 8) with nanodroplet cavitation reagent MegaShear (Triangle Biotechnology) using Covaris LE220-plus ultrasonicator. Samples were sonicated enough to produce DNA fragments ∼200–500 bp. DNA fragments were incubated with H3K9me3 (Abcam8898) and Dynabeads Protein G (Invitrogen cat. #10004D) overnight at 4°C with overnight rotation. Immunoprecipitation was performed with magnetic strip and washed with ChIP IP buffer (50  mM HEPES/KOH pH 7.5, 300 mM NaCl, 1 mM DETA, 1% Triton X100, 0.1% DOC, 0.1% SDS) twice. The IP beads were treated with Proteinase K (Invitrogen) overnight at 65°C. The supernatants were collected the next day and purified using Qiagen MinElute PCR Purification Kit.

RT-qPCR were performed using SYBR Green Master Mix (Rox) on ViiA 7 system (Applied Biosystem). Each time point was measured in at least biological duplicate and the RT-qPCR measurements were quantified from technical quadruplicates. Samples were analyzed using ΔΔCt method and normalized against an intergenic control region or normalized to a housekeeping gene. The qPCR primers are listed in Supplementary Table S2.

### Statistical analysis and graph generation

Statistical analysis was performed using paired Student’s t test on Excel, and statistical significance was determined with alpha = 0.05. Graphs were generated using GraphPad Prism software.

### Monte Carlo simulation methods

The simulated system consists of an unmethylated chromatin fiber (102 nucleosomes) and 102 copies of each protein—HP1, G9a, SUV39H, SETDB1, and a generic lysine demethylase (KDM)—corresponding to a putative cellular concentration level [on the order of 10 µM (71)]. The particle-based chromatin model, the Monte Carlo simulation method, and all simulation settings (box, voxels, time step, and output) were used as previously described (33). The time step of 1 μs was chosen to maximize the sampling rate while keeping the particle displacements within the system's resolution, that is, by avoiding overstretching the particle-to-particle tethers. Diffusion rates for the molecular objects were estimated from the bulk measurements of protein diffusion in a cellular compartment (66–68) (neither of the studies involved HP1, methyltransferases, or nucleosomes). The diffusion coefficient of 0.550 μm^2^ s^−1^ for HP1 in our simulations proved to be consistent with the experimental data obtained specifically for HP1 diffusion coefficient (69, 70). The starting configuration consisted of an unmethylated (except two permanently methylated central nucleosomes), randomly folded chromatin and randomly scattered proteins. Seven distinct systems were simulated (25 randomly initialized replicas per system). The simulated systems included a WT 1 and 6 knockdown systems where the concentration of each HMT was reduced to 75 and 50% of their respective WT concentrations to mimic the experimental knockdown conditions. Supplementary Tables S3 and S4 provide a full set of parameters utilized in the simulations (see below a detailed explanation of how the parameters used are linked to experimental data).

#### Proteins, protein–protein interactions, and enzymatic reactions

Four new types of proteins (G9a, SUV39H, SETDB1, and KDM) were introduced in this study (see Supplementary Tables S3–S5 for the respective parameters). Each HMT was modeled as a chain of two particles—a chromatin- or HP1-binding anchor (*bind*) and a catalytic SET domain (*cat*) (see next paragraph for the rationale of having distinct tethered *bind* and *cat* particles). The *bind* and *cat* particles of the HMTs were allowed to freely move within a distance range between *R_bind_* + *R_cat_* and *D_std_*, where *R_bind_* and *R_cat_* (equals to 1.5 nm each) are radii of *bind* and *cat* domains, respectively, and *D_std_* (equals to 4 nm) is the standard distance maintained by a disordered linker connecting *bind* and *cat* with an exponential damping beyond *D_std_*. The damping multiplier *k* in *exp(-kΔd)* was set to 0.3. We made sure that the time step of 1 μs would not result in a systematic overstretching of the *bind*-*cat* tether. PDF for *bind*-*cat* distances over the ensemble of simulations is typical of a random polymer. KDM is a one-particle object with a radius of 1.5 nm and a mass of 130 kDa.

The protein–protein interactions for the four enzymes include binding of the *bind* particle of each HMT to HP1 and binding of KDM to methylated H3K9. The interactions in the simulated system occur as previously described (33) and are governed by the association (*p*_a_) and dissociation (*p*_d_) probabilities that parameterize stochastic processes “carried” by the interacting particles (see Tables S4 and S5 for a full list of interaction parameters). The *p*_a_ and *p*_d_ parameters were inferred from experimental data based on the current knowledge of how the three HMTs bind to chromatin. The major putative chromatin-binding mechanism involves HMTs binding to the HP1 CSD [with a *K_D_* on the order of 10 µM (71, 83, 84)] whose CD may concurrently bind to methylated H3K9 (39, 46, 85–88). This mechanism is successfully exploited in our chromatin in vivo experiments (25, 33, 89). HMT-CSD binding occurs either directly (25, 39, 87, 88) or is mediated by additional HP1 protein that might be either a member (along with HMT) of a large epigenetic complex, such as E2E6 (90), or be anchored (through its CD) to the HMT's methylated lysine (74). Additionally, G9a and SETDB1 are able to bind to methylated chromatin through their Kme-binding modules. Those known mechanisms were embedded in our model through interactions of the HMTs’ *bind* and *cat* particles with HP1-CSD and H3K9. Experimental kinetic data were used to calibrate the *p*_a_ and *p*_d_ parameters for chromodomain binding to H3K9 and chromoshadow dimerization. As an example, *p*_d_ and *p*_a_ values were determined as *p*_a_ = *p*_d_*[*F*]*k_on_/*k*_off_, with [*F*] equal to the free binding partner concentration and where *k*_on_/*k*_off_ was equal to the experimentally determined association and dissociation rates as previously described, respectively (33, 91). HP1 on heterochromatin *k*_on_ and *k*_off_ were measured as, respectively, ∼1 mM^−1^s^−1^ and ∼4 s^−1***^ (91). Relevance of the *p*_a_ and *p*_d_ parameters was validated by calculating the equilibrium binding constants from the simulation trajectories. The *K*_d_ values obtained were consistent with the experimental data (on the order of 5–15 mM). The respective individual *p*_a_ and *p*_d_ values (see Supplementary Table S5 for a full set of *p*_a_ and *p*_d_ values) were tuned to yield HMT-chromatin-binding constants to match experimentally observed apparent binding data (71). The *p*_a_ and *p*_d_ values were verified for Suv39H for which experimental data on interactions with chromatin are available (71) [by comparing the respective calculated and experimental binding constants that are both on the order of ∼10 µM (71)]. The *p*_a_ and *p*_d_ values for the generic KDM [the most likely H3K9 demethylases are KDM1A/B and KDM4 (72, 76, 92)] were chosen to reproduce *K_i_* for the substrate on the order of 10 µM (77).

The lysine methylation reaction on H3K9 was modeled as a stochastic process linked to an HMT's *cat* particle. For the reaction to occur, three conditions must be met: (i) the HMT's *bind* particle must be anchored to chromatin [this binding event sets the *cat* particle into an active state, e.g. through conformational rearrangement (93)], (ii) the *cat* particle must bind to H3K9me0/1/2, and (iii) the random number from the *cat*'s stochastic process must be smaller than the respective *p*_t_ value, the transform probability specific to a particular HMT/methylation state pair (see Supplementary Table S6 for a full set of *p*_t_ values). When all the above conditions concur, the H3K9 particle bound to the HMT's *cat* particle is switched into a higher methylation state (e.g. from me0 to me1). After the H3K9 transformation occurred, the HMT's *cat* particle is set into an inactive state to prevent a possible immediate H3K9 transform into the next methylation state [to mimic the real-life process where a new enzymatic reaction would require some preparation time, e.g. co-factor exchange and necessary conformational rearrangements (93)]. The individual *p*_t_ parameters were calibrated to reflect the enzyme turnover times (on the order of 10–10^2^ s) (72, 73), as well as methylation-specific rates. The highest *p*_t_ values were set for the primary enzyme activities [mono-methylation for G9a (74), di-methylation for SETDB1 (73), and tri-methylation for SUV39H (75)]. Reduced rates were parameterized for secondary reactions according to the available data (72, 73, 75) (see Supplementary Table S6 for a full list of *p*_t_ values). The demethylation reaction catalyzed by KDM differs in that KDM is a one-particle object, and hence, demethylation requires only two conditions to be met: (i) binding to a methylated H3K9 and (ii) a value from stochastic process lower than the respective *p*_t_. Experimental data for KDM1A/B and KDM4C were used to calibrate the demethylation *p*_t_ (76, 77).

## Supplementary Material

pgad062_Supplementary_Data

## Data Availability

All data required for main findings of this manuscript are included in the article and Supplementary material. Monte Carlo simulation model source code for MATLAB analysis is publicly deposited at http://dx.doi.org/10.17632/ztz34f5st2.1
