# Supplementary material for: Relationship between lysine methyltransferase levels and heterochromatin gene repression in living cells and in silico
Source: PNAS Nexus. 2023 Mar 7;2(4):pgad062. doi: 10.1093/pnasnexus/pgad062 (PMC10069619; doi:10.1093/pnasnexus/pgad062)
Supplement: pgad062_Supplementary_Data [file pgad062_supplementary_data.zip › PNASNEXUS-PNASNEXUS-2022-01153-T-s01.docx]

**Table S1.** dCas9 gRNA sequences targeting SetDB1

| Position relative to the transcriptional start site | gRNA sequence |
| --- | --- |
| -64 | CTACCGTCGGAGAGGCCGGA |
| -77 | TGGTCCCTTCGGGCTACCGT |
| -117 | ACTCTGGCGCCCGACCGCAA |
| -135 | GTTGTGGGGAGGACGGACTG |
| -211 | AGAGCTTCCATAAATGACTC |
| NT | GCGAGGTATTCGGCTCCGCG |

**Table S2** PCR primers used in this paper

| Primer Name | Primer sequence |
| --- | --- |
| SetDB1 RT-qPCR Forward Primer | GCCCACAGAGATCATTGAGATT |
| SetDB1 RT-qPCR Reverse Primer | CTTTTGGAGTTCTGCTCCCA |
| GAPDH RT-qPCR Forward Primer | CCAATGTGTCCGTCGTGGATCT |
| GAPDH RT-qPCR Reverse Primer | GTTGAAGTCGCAGGAGACAACC |
| ChIP qPCR 169 Forward | TGCTCCTCCACCCACCCA |
| ChIP qPCR 169 Reverse | AATCCCACCCTCTAGCCTTG |
| ChIP qPCR 489 Forward | GCGCACCATCTTCTTCAAGG |
| ChIP qPCR 489 Reverse | AGCTCGATGCGGTTCACCA |
| ChIP qPCR 739 Forward | GCACTTCTCTGGGGTCTCTG |
| ChIP qPCR 739 Reverse | CAGAGTTTAGAGGCTCTACAC |
| ChIP qPCR IGR Forward | CCCTATTACTTCGTGTCTGTCG |
| ChIP qPCR IGR Reverse | AGTCAGAGAGGCCAAGAACA |

**Table S3.** Individual particles’ parameters utilized in the simulations.

| Particle | Radius (nm) | Mass (kDa) |
| --- | --- | --- |
| Nucleosome core | 2.5 | 200 |
| H3_core | 1 | 12 |
| H3_K9 | 1 | 1 |
| H3_K9me3 | 1 | 1 |
| HP1_cd | 1.5 | 8 |
| HP1_csd | 1.5 | 8 |
| HMT_bind | 1.5 | 70 |
| HMT_cat | 1.5 | 70 |
| KDM | 1.5 | 100 |

**Table S4.** Parameters of particle-particle tethers utilized in the simulations.

| Particle 1 | Particle 2 | D_min_ (nm)* | D_max_ (nM) | k** |
| --- | --- | --- | --- | --- |
| Nucleosome core | Nucleosome core | 13 | 17 | 0.5 |
| HP1_cd | HP1_csd | 2 | 4 | 0.3 |
| H3_core | H3_K9 | 1 | 4 | 0.3 |
| H3_core | H3_K9me3 | 1 | 4 | 0.3 |
| HMT_bind | HMT_cat | 3 | 5 | 0.833 |

*Minimal and maximal particle-particle distances allowed without a penalty applied.

**as well as the “spring-like” constant (k).

**Table S5.** Association/dissociation probabilities of the interacting particles utilized in the simulations.

| Particle 1 | Particle 2 | Reaction | Probability |
| --- | --- | --- | --- |
| H3_K9me3 | HP1_cd | Association | 0.001 |
| H3_K9me2 | HP1_cd | Association | 0.0001 |
| HP1_csdA | HMT#_bind | Association | 0.001 |
| H3_K9 | HMT1*_catA****** | Association | 0.00001 |
| H3_K9me1 | HMT1_catA | Association | 0.000001 |
| H3_K9me2 | HMT1_catA | Association | 0.0000001 |
| H3_K9 | HMT2_catA | Association | 0.000001 |
| H3_K9me1 | HMT2_catA | Association | 0.00001 |
| H3_K9me2 | HMT2_catA | Association | 0.000001 |
| H3_K9 | HMT3_catA | Association | 0.0000001 |
| H3_K9me1 | HMT3_catA | Association | 0.000001 |
| H3_K9me2 | HMT3_catA | Association | 0.00001 |
| HP1_csdA | HP1_csdA | Association | 0.00001 |
| H3_K9me# | KDM | Association | 0. 0001 |
| H3_K9me3 | HP1_cd | Dissociation | 0.0000001 |
| HP1_csd | HP1_csd | Dissociation | 0.0000001 |
| HP1_csd | HMT#_bind | Dissociation | 0.01 |
| HP1_csdA | HMT#_bindA | Dissociation | 0.01 |
| H3_K9# | HMT#_catA | Dissociation | 0.02 |
| H3_K9# | KDM | Dissociation | 0.01 |

***HMT1 represents G9a, HMT2 represents SetDB1, and HMT3 represents Suv39H in the simulation parameters.**

****Active (A) and inactive (U) *cat* particle states**

**Table S6.** Particle transformation and state change probabilities of the interacting particles utilized in the simulations.

| Substrate | Pre-*cat*-state | Reaction | Probability | Product | Post-*cat*-state |
| --- | --- | --- | --- | --- | --- |
| H3_K9 | HMT1_catA* | Methylation | 0.01 | H3_K9me1 | HMT1_catU* |
| H3_K9me1 | HMT1_catA | Methylation | 0.0001 | H3_K9me2 | HMT1_catU |
| H3_K9me2 | HMT1_catA | Methylation | 0.0001 | H3_K9me3 | HMT1_catU |
| H3_K9 | HMT2_catA | Methylation | 0.0001 | H3_K9me1 | HMT2_catU |
| H3_K9me1 | HMT2_catA | Methylation | 0.01 | H3_K9me2 | HMT2_catU |
| H3_K9me2 | HMT2_catA | Methylation | 0.0001 | H3_K9me3 | HMT2_catU |
| H3_K9 | HMT3_catA | Methylation | 0.0001 | H3_K9me1 | HMT3_catU |
| H3_K9me1 | HMT3_catA | Methylation | 0.0001 | H3_K9me2 | HMT3_catU |
| H3_K9me2 | HMT3_catA | Methylation | 0.01 | H3_K9me3 | HMT3_catU |
| H3_K9me# | KDM | Demethylation | 0. 01 | He_K9(-1) | KDM |
| HMT#_bindA | HMT*_cat | State | 0.01 | HMT#_bindA | HMT#_catA |
| HMT#_bindA | HMT#_catU | State | 0.01 | HMT#_bind | HMT#_cat |
| HMT#_bindA | HMT#_catA | State | 0.00001 | HMT#_bind | HMT#_cat |
| HP1_cdA | HP1_csd | State | 0.01 | HP1_cdA | HP1_csdA |
| HP1_cdA | HP1_csdA | State | 0.000001 | HP1_cd | HP1_csd |

*Active (A) and inactive (U) *cat* particle states
