# Supplementary material for: Relationship between lysine methyltransferase levels and heterochromatin gene repression in living cells and in silico
Source: PNAS Nexus. 2023 Mar 7;2(4):pgad062. doi: 10.1093/pnasnexus/pgad062 (PMC10069619; doi:10.1093/pnasnexus/pgad062)
Supplement: pgad062_Supplementary_Data [file pgad062_supplementary_data.zip › PNASNEXUS-PNASNEXUS-2022-01153-T-s02.pdf]

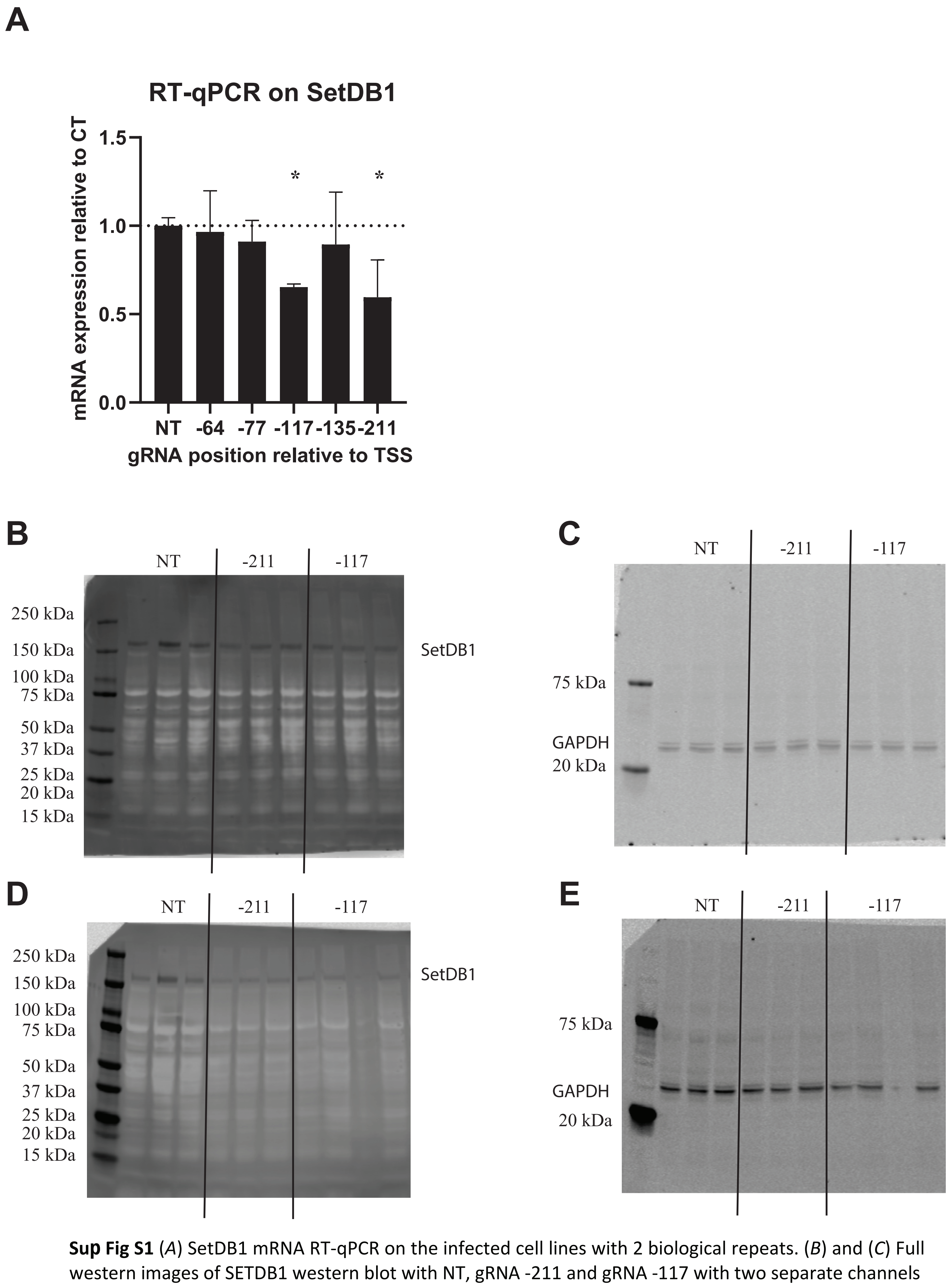

Sup Fig S1 (*A*) SetDB1 mRNA RT-qPCR on the infected cell lines with 2 biological repeats. (*B*) and (*C*) Full western images of SETDB1 western blot with NT, gRNA -211 and gRNA -117 with two separate channels for different antibodies. (*D*) and (*E*) technical repeat of western blot of (*B*) and (*C*).

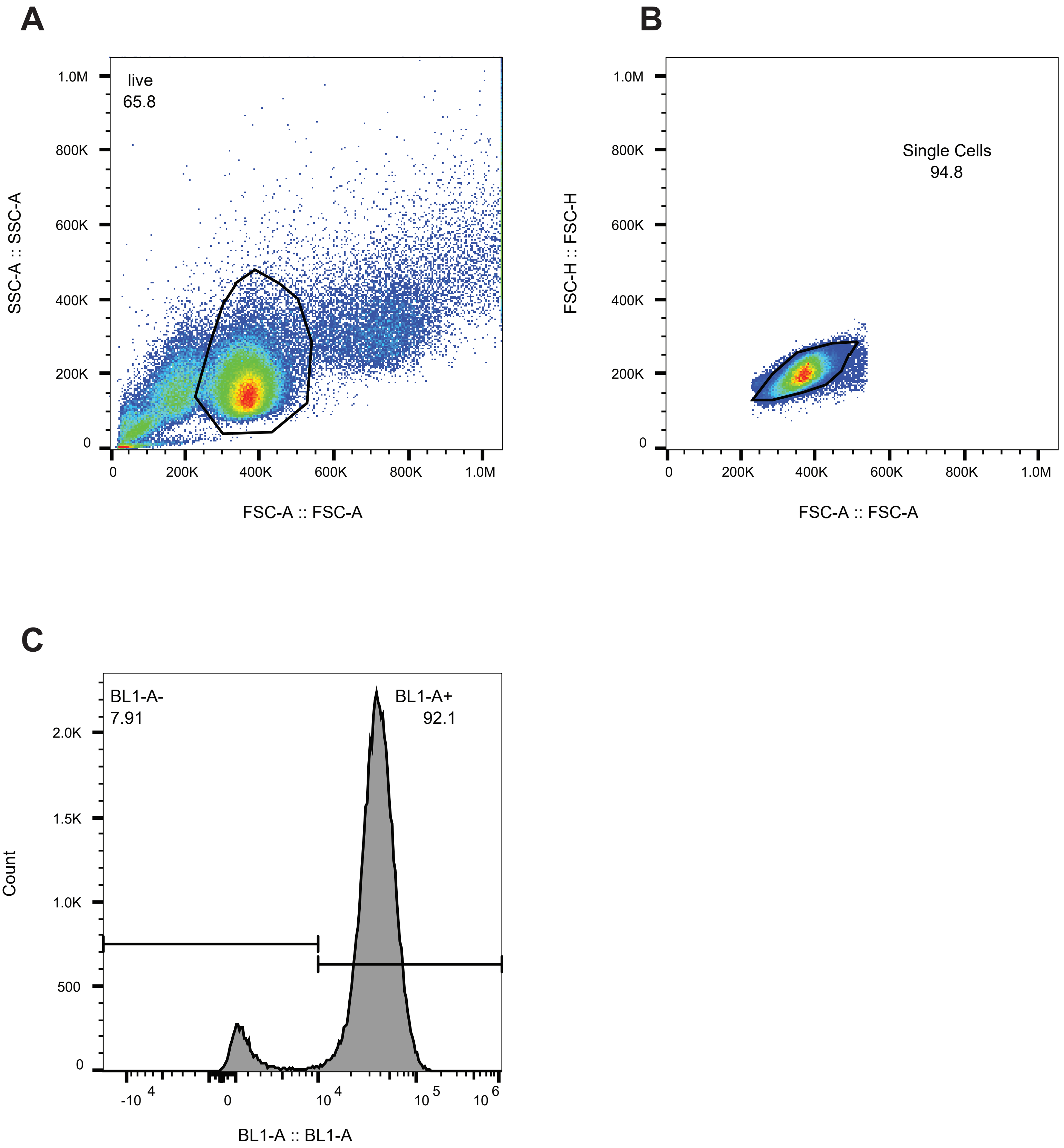

Sup Fig S2, (*A*)flow cytometry forward vs sideway scatter area used to separate the live cells and dead cells. The boxed cell population is considered live cells. (*B*) flow cytometry forward scatter area vs forward scatter height used to identify single cells. The boxed cell singlet population is used for eGFP analysis. (*C*) the 0 days of HP1α recruitment cell population is used to identify eGFP positive population, the majority of the cell has high eGFP signal.

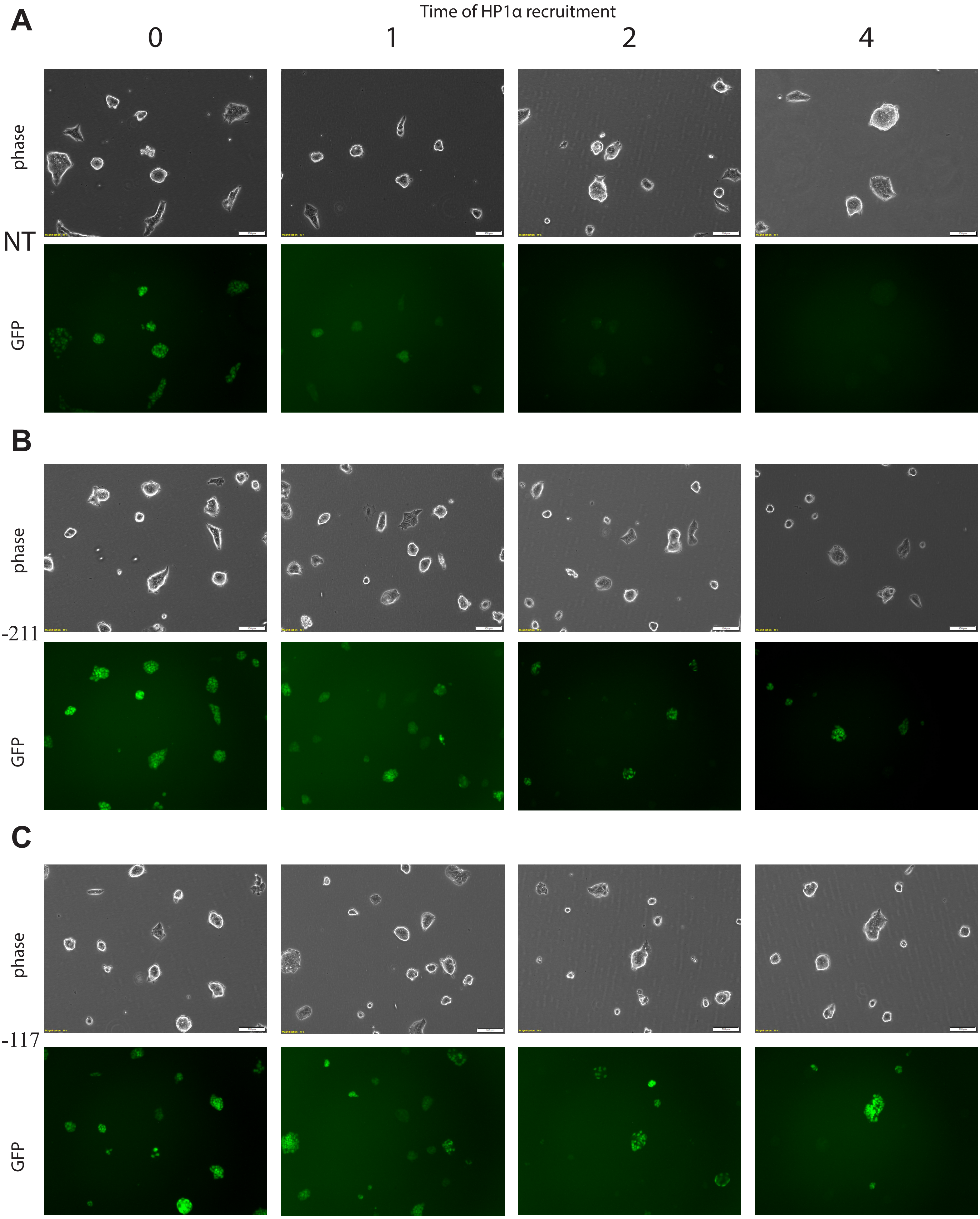

Sub Fig S3, (*A*) individual cell images of NT cell lines under different HP1α recruitment time in Phase and GFP Channel. (*B*) individual cell images of -211 gRNA infected cell lines under different HP1α recruitment time in Phase and GFP Channel. (*C*) individual cell images of -117 gRNA infected cell lines under different HP1α recruitment time in Phase and GFP Channel.

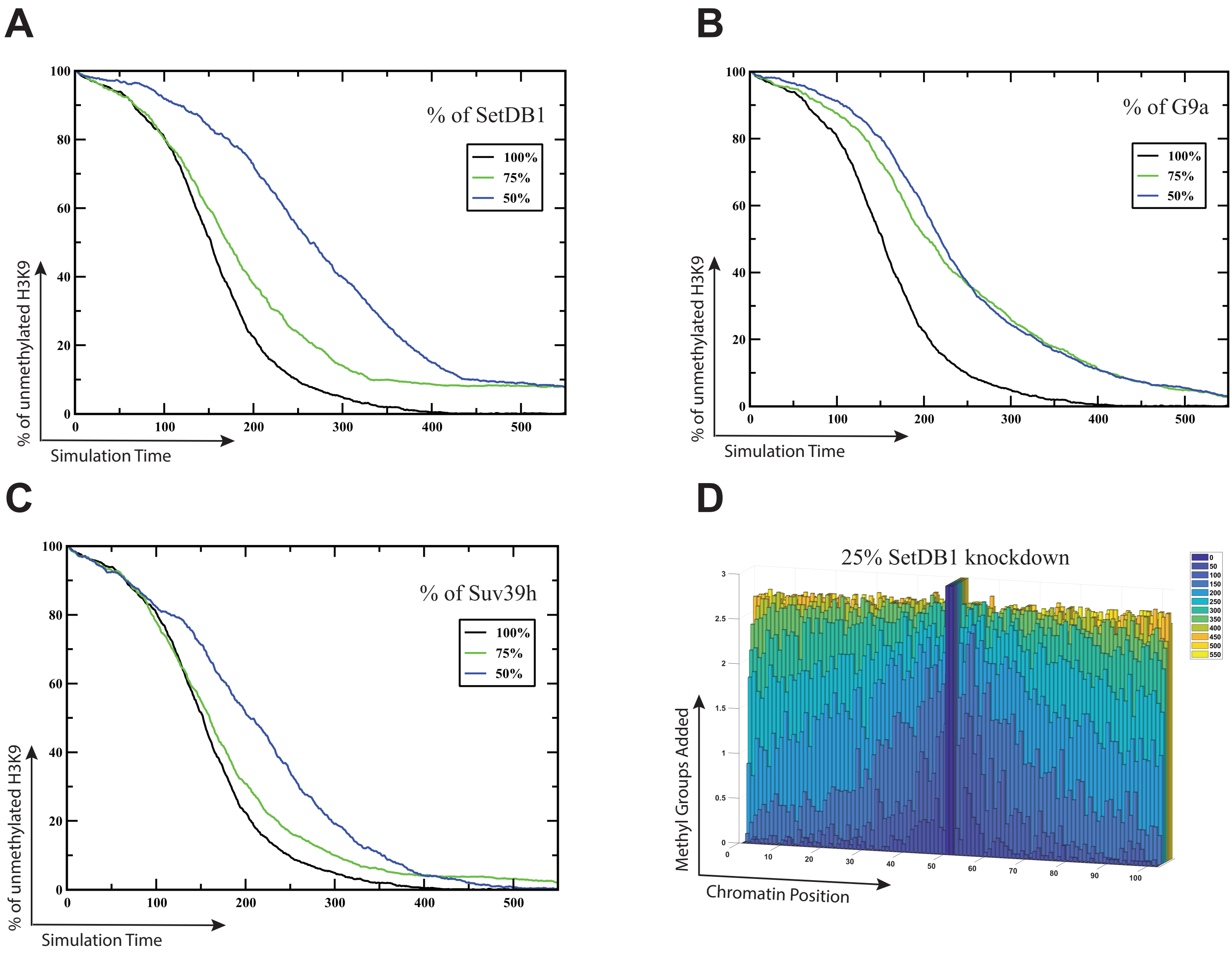

Sup Fig S4, % of overall unmethylated H3K9 tails over the simulation time under different SetDB1 knockdown conditions for (*A*)**,** G9a for (*B*) and Suv39h for (*C*). (*D*)
